# Early maladaptive schemas in adult survivors of interpersonal trauma: foundations for a cognitive theory of psychopathology

**DOI:** 10.3402/ejpt.v7.30713

**Published:** 2016-09-16

**Authors:** Thanos Karatzias, Sally Jowett, Amelie Begley, Suzanne Deas

**Affiliations:** 1 School of Health & Social Care, Edinburgh Napier University, Edinburgh, UK; 2 NHS Lothian, Rivers Centre for Traumatic Stress, Edinburgh, UK; 3 NHS Fife, Clinical Psychology Department, Lynebank Hospital, Dunfermline, UK

**Keywords:** Interpersonal trauma, schemas, psychopathology, PTSD

## Abstract

**Background:**

Although the association between psychological trauma and early maladaptive schemas (EMS) is well established in the literature, no study to date has examined the relationship of EMS to PTSD and psychopathologies beyond depression and anxiety in a sample of adult survivors of interpersonal trauma. This information may be useful in helping our understanding on how to best treat interpersonal trauma.

**Objective:**

We set out to investigate the association between EMS and common forms of psychopathology in a sample of women with a history of interpersonal trauma (*n*=82). We have hypothesised that survivors of interpersonal trauma will present with elevated EMS scores compared to a non-clinical control group (*n*=78). We have also hypothesised that unique schemas will be associated with unique psychopathological entities and that subgroups of interpersonal trauma survivors would be present in our sample, with subgroups displaying different profiles of schema severity elevations.

**Method:**

Participants completed measures of trauma, psychopathology, dissociation, self-esteem, and the Young Schema Questionnaire.

**Results:**

It was found that survivors of interpersonal trauma displayed elevated EMS scores across all 15 schemas compared to controls. Although the pattern of associations between different psychopathological features and schemas appears to be rather complex, schemas in the domains of Disconnection and Impaired Autonomy formed significant associations with all psychopathological features in this study.

**Conclusions:**

Our findings support the usefulness of cognitive behavioural interventions that target schemas in the domains of Disconnection and Impaired Autonomy in an effort to modify existing core beliefs and decrease subsequent symptomatology in adult survivors of interpersonal trauma.

**Highlights of the article:**

- Interpersonal trauma survivors are distinguished primarily by a generalised elevation of their maladaptive schemas, rather than a unique schema profile comprised of specific schemas.
- A strong profile was formed in the domains of 'Disconnection' and 'Impaired Autonomy', where both presented with strong associations with psychopathological entities.
- CBT interventions should target schemas such as 'Vulnerable to Harm', to alleviate mental health distress in people with interpersonal trauma.

Interpersonal trauma includes any type of traumatic event wherein another person causes the trauma. Typical examples include childhood maltreatment, child abuse, rape, assault, domestic abuse, emotional abuse, and neglect. Interpersonal trauma survivors tend to have higher rates of psychopathology such as posttraumatic stress disorder (PTSD) (e.g., Forbes et al., 2012) or depression (e.g., Iverson et al., 2011) compared to survivors of trauma of a non-interpersonal nature. History of interpersonal trauma, occurring in childhood or adulthood, has also been associated with a high risk of developing a wide range of psychiatric disorders including not only posttraumatic stress and mood disorders but also dissociative, addictive, eating, and personality disorders (Maniglio, 2009; Schumm, Briggs-Phillips, & Hobfoll, 2006). Because interpersonal trauma is associated with various disorders and complex constellations of symptoms, this has led to a need to look for underlying cross-diagnostic mental constructs that could enhance our understanding of interpersonal trauma sequelae and subsequently inform appropriate interventions. Schema theory offers a useful framework to study the complex and adverse outcomes associated with interpersonal trauma.

Schema theory integrates the assumptions of psychodynamic, cognitive-behavioural, and attachment theories. A “schema” comprises subjective constructs that contain a broad pattern of memories, emotions, and cognitions that guide behaviour. Schemas can determine how one perceives others, the self, and relations with others. Schemas originate in early childhood or adolescence and become increasingly stable over time unless significant corrective experiences are made. However, cultural and temperament can also contribute to schema activation (Young, Klosko, & Weishar, 2003). It has been proposed that early maladaptive schemas (EMS) can develop as a consequence of harmful interpersonal experiences. Interpersonal trauma violates basic needs for safety, guidance, and affection, and thus can be a source of EMS. Young et al. (2003) proposed that schemas of Mistrust/Abuse, Defectiveness/Shame, or Vulnerability to Harm result from early traumatic or victimisation experiences of an interpersonal nature. Previous evidence suggests that maladaptive schemas are linked to a variety of psychological disorders commonly presented in people with histories of interpersonal trauma including adult depression and anxiety (O'Dougherty Wright, Crawford, & Del Castillo, 2009), social phobia (Pinto-Gouveia, Castilho, & Cunha, 2006), eating disorder symptomatology (e.g., Waller, Kennerley, & Ohanian, 2007), personality disorders (Petrocelli, Glaser, Calhoun, & Campbell, 2001), self-harm behaviours (Castille et al., 2007), attachment difficulties (Mason, Platts, & Tyson, 2005), interpersonal conflict (Messman-Moore & Coates, 2007), and general psychological distress (Schmidt & Joiner, 2004). There is also evidence to suggest that EMS may mediate the relationship between early adversity (i.e., through poor parenting, neglect, abuse) and the later development of depression and anxiety psychopathologies (Harris & Curtin, 2002; Lumley & Harkess, 2007; McGinn, Cukor, & Sanderson, 2005).

With regard to the association between EMS and PTSD, Price (2007), in a sample of male and female health workers with PTSD following work-related trauma, found that four schemas (Defectiveness, Dependency, Enmeshment, and Failure) significantly predicted PTSD status. Although this demonstrates that specific schemas are associated with PTSD, the authors did not differentiate between interpersonal and non-interpersonal trauma. Harding, Burns, and Jackson (2012), in a sample of 127 female child sexual abuse (CSA) survivors, identified three distinctive clusters of schema elevation. Women in the cluster with the highest maladaptive schema scores reported the most severe PTSD symptoms. Schemas of Mistrust/Abuse, Vulnerability to Harm, and Emotional Deprivation contributed most to distinguishing women with a diagnosis of PTSD. Therefore, interpersonal trauma history appears to be associated with current schema presentation, and these schemas may in turn mediate a relationship between the trauma and current psychological distress.

In this study, we sought to extend previous research by examining the association between EMS and psychopathology in adult survivors of interpersonal trauma, using a broader range of measures than the preceding literature. This was achieved by examining the relation between EMS and various psychopathological entities commonly found in adult survivors of interpersonal trauma, including PTSD, anxiety, depression, general distress, dissociation, and pervasive low self-esteem. No study to date has examined the relationship of EMS to PTSD and psychopathologies beyond depression and anxiety in a sample of adult survivors of interpersonal trauma.

On the basis of previous theoretical and empirical literature supporting, the association between interpersonal trauma and EMS (e.g., Harding et al., 2012; Price, 2007) in people who have experienced adverse life events, we have hypothesised that:

1. Survivors of interpersonal trauma will present with elevated EMS compared to a non-clinical control group from the general population.
2. Different schemas will be associated with different psychopathological entities; specifically abuse-related schemas such as Vulnerability to Harm, Mistrust/Abuse, and Defectiveness/Shame will predict higher levels of different types of psychopathology. This will explain the considerable heterogeneity with regard to clinical presentations of adult survivors of interpersonal trauma.
3. Different subgroups of interpersonal trauma survivors will be present in our sample, with subgroups displaying different schema severity elevations.
4. Those with elevated EMS scores will present with more severe traumatic, general, and dissociative psychopathology as well as low self-esteem scores compared to those with moderate EMS severity scores.

## Methods

### Participants

Two groups of participants were recruited for the study; those who had experienced interpersonal trauma and a control group.

#### Interpersonal trauma group

Participants with a history of interpersonal trauma were a consecutive series of patients (*n*=82) from the waiting lists of outpatient psychological therapy clinics in five National Health Service (NHS) Boards across Scotland. Trauma history was assessed in the clinical group by means of a clinical interview. Inclusion and exclusion criteria are described as follows: *Inclusion Criteria*: Female service users with a history of interpersonal trauma (CSA, child neglect, physical abuse, assault, and domestic violence) and subsequent psychological distress (i.e., traumatic symptomatology, dissociation, self-esteem, and general distress) in the waiting list of psychological therapy services, being willing to participate voluntarily, being able to give written informed consent, and aged between 18 and 65 years old.

#### Control group

The control group was originally recruited for another study on disordered eating (Deas, Power, Collin, Yellowless, & Grierson, 2011) and allowed a comparison to healthy females in the general population. The control group consisted of female students currently in an undergraduate psychology degree in a Scottish university. History of trauma was not available. Exclusion criteria were: male gender, under 18 or over 65 years of age, and previous or current input from services for concerns over eating or depression/anxiety. Of the 120 female students approached, 82 were participated (response rate of 68%). Four were excluded on the basis of current eating concerns (*N*=1) and depression/anxiety (*N*=3). This left a total of 78 female students in the control group.

### Procedure

Following consent to participate and confirmation of inclusion/exclusion criteria, participants in the clinical group completed paper-and-pencil self-report measures of demographic information and psychopathology. All assessments were completed in a single interview. Ethical approval for the study involving the clinical sample was obtained from Edinburgh Napier University and the Integrated Research Application System (IRAS), prior to commencement of the research process. For the study involving the control group, ethical approval was obtained from IRAS, The Priory Hospital Glasgow, and the University of Stirling's Psychology Department Ethics Committee.

### Measures

*Basic demographics* include age, gender, and educational attainment for the clinical group. For the control group, data on the Young Schema Questionnaire (YSQ) (Young & Brown, 1994a) have been used as a part of this project. A number of self-report measures were also completed by the participants in the clinical group as follows:

#### The Young Schema Questionnaire-Short Form

The YSQ is a 75-item self-rated scale of EMS. Participants respond on a 6-point scale, ranging from 1 (“completely untrue of me”) to 6, (“describes me perfectly”), where a higher score indicates more presence of maladaptive schemas (Young & Brown, 1994b). Items are organised in 15 subscales that describe different EMS, and each schema subscale contains five items. There are five domains to organise related schemas.

The Disconnection and Rejection domain is comprised of schemas relating to themes of Abandonment (“I find myself clinging to people I'm close to, because I'm afraid they'll leave me”), Mistrust/Abuse (“I am quite suspicious of other people's motives”), Emotional Deprivation (“For much of my life, I haven't felt that I am special to someone”), and Defectiveness/Shame (“I am too unacceptable in very basic ways to reveal myself to other people”). The Impaired Autonomy domain is comprised of schemas relating to Social Isolation (“I don't fit in”), Dependence (“I do not feel capable of getting by on my own in everyday life”), Vulnerability to Harm (“I worry about being attacked”), Enmeshment (“I often feel that I do not have a separate identity from my parent or partner”), and Failure (“I'm incompetent when it comes to achievement”). The Impaired Limits domain is comprised of schemas relating to Entitlement (“I hate to be constrained or kept from doing what I want to do”) and Insufficient Self-Control (“I can't seem to discipline myself to complete routine or boring tasks”). The Other-Directedness domain is comprised of schemas relating to Subjugation (“In relationships, I let the other person have the upper hand”) and Self-Sacrifice (“Other people see me as doing too much for others and not enough for myself”); and finally, the Overvigilance and Inhibition domain is comprised of schemas relating to Emotional Inhibition (“I control myself so much that people think that I am unemotional”) and Unrelenting Standards (“I must meet all my responsibilities”).

Previous studies have demonstrated strong internal consistency, reliability, validity, and factor structure of the YSQ (Hoffart et al., 2005; Rijkeboer & Van den Bergh, 2006). Furthermore, the parallels found between the YSQ and YSQ-SF in reliability, validity, and predictive abilities indicate that the short form can be used with confidence (Stopa, Thorne, Waters, & Preston, 2001).

#### PTSD Checklist-Civilian Version

The PTSD Checklist-Civilian Version (PCL-C) is a self-report 17-item standardised questionnaire which assesses posttraumatic symptoms (e.g., intrusive memories) over the last week (Blanchard, Jones-Alexander, & Buckley, 1996). Participants respond on a 5-point scale, ranging from “not at all” to “extremely” for how much the specific symptom was a problem to them over the past month. An overall score and sub-scores for re-experience, avoidance, and hyperarousal subscales are provided. A higher score indicates higher traumatic symptomatology. The PCL-C has demonstrated good internal consistency (*α*=0.94) and test–retest reliability (*r*=0.66) and, in comparison to other PTSD measures, shows superior patterns of discriminant and convergent validity (Conybeare, Behar, Soloman, Newman, & Borkovec, 2012).

#### The Symptom Checklist-90

The Symptom Checklist-90 (SCL-90) is a standardised self-report instrument for measuring general psychopathology. There are also subscales that capture aspects of interpersonal sensitivity and emotional dysregulation (e.g., Hostility) (Derogatis, 1979). It contains 90 problem items rated on a 5-point Likert scale and comprises nine sub-scales: Somatisation, Obsession–Compulsion, Interpersonal Sensitivity, Depression, Anxiety, Hostility, Phobic Anxiety, Paranoid Ideation, and Psychoticism. Seven additional items do not belong to any sub-scale. The nine sub-scales can be combined into the Global Severity Index (GSI), which is a global index of distress. A higher score indicates higher psychological distress. The reliability of the SCL-90 is regarded as satisfactory across the literature (Prinz et al., 2013); with internal consistency found to range between *r*=0.77–0.90 (Derogatis, 1977), test–retest reliability ranging from 0.78 to 0.90 (Derogatis & Melisaratos, 1983). There is also strong support for the validity of the SCL-90; however, there are inconsistent findings with regard to its proposed dimensional structure (Holi, 2003).

#### Dissociative Experiences Scale

Dissociative Experiences Scale (DES) is a 28-item self-report measure of the frequency of a number of dissociative symptoms (e.g., gaps in awareness, depersonalisation) (Bernstein & Putnam, 1986). Respondents rate the percentage of time (i.e., 0–100%) that they experience each symptom/item. No time frame for assessment is specified. A higher score indicates higher levels of dissociation. The DES has demonstrated strong psychometric properties in terms of reliability, test–retest reliability, internal consistency, predictive validity, and convergent validity (Holtgraves & Stockdale, 1997).

#### Rosenberg self-esteem scale

Rosenberg self-esteem scale (RSES) is a 10-item standardised self-report measure of self-esteem. Respondents report feelings about their self, using a 4-point response format (strongly agree, agree, disagree, and strongly disagree) (Rosenberg, 1965). No time frame for assessment is specified. A higher score indicates higher self-esteem. Various analyses on the RSES have found the scale to have good internal consistency and test–retest reliability (Blascovich & Tomaka, 1991; Robins, Trzesniewski, Tracy, Gosling, & Potter, 2002).

### Data analysis

SPSS 21 was used for data analysis. Means (standard deviations, SDs) were calculated for all continuous variables and frequencies (%) for all categorical variables. *T*- and *F*-tests were used for comparisons between groups. Associations between variables were explored by means of Spearman correlations. To answer question b (i.e., different schemas will be associated with different psychopathological entities), linear forward regression analyses were conducted to investigate the association between individual YSQ sub-scales and the pathology measures of PCL-5, SCL-90, DES, and RSES. Due to the high number of variables, a corrected *p*-value of 0.025 was used in order to reduce the likelihood of a Type I error. The following assumptions were checked prior to conducting regression analysis. Normally distributed residuals and homoscedasticity were confirmed through plotting these as part of the regression analyses. The assumption of linearity was met through plotting correlations between dependent and independent variables on scatterplots and observing linear relationships for all. All outliers were identified and cleaned from the dataset prior to analysis. Our analyses also tested for the assumption of multicollinearity. As part of the regression, the variance inflation factor (VIF) was calculated to identify multicollinearity amongst the YSQ predictor variables. This analysis showed that the VIF ranged between 1.202 and 3.487, demonstrating some collinearity but not to a degree where the regression results would be compromised. Due to the nature of the variables, the assumption of normal distribution was not met. However, further inspection of the Q–Q plots demonstrated that the residuals for each variable were normally distributed and, as the data were all skewed in the same direction for each variable, the regression analyses were robust enough to provide a reliable outcome.

Cluster analyses were computed on the YSQ subscales in order to identify unique schema profiles within the trauma survivor group in line with question c. Specifically, a k-means cluster analysis was adopted to identify two groups, low EMS and high EMS. Although previous research identified three clusters (Harding et al., 2012), our sample is a third smaller and so two groups elicit a more meaningful difference between low and high scores. As cluster analysis utilises the distance from the mean, it requires that all scores are standardised in order to prevent data distortion; as only the YSQ data were included, all of the scores were measured on the same scale, and thus, data were standardised.

## Results

### Sample characteristics

Demographic data for the clinical sample are shown in Table 1. Means (SDs) of clinical scales including PCL-C, SCL-90, DES, and RSES are presented in Table 2.

**Table 1:** Demographic data for the clinical sample

	*M* (SD) or *n* (%) *n*=82
Age	40.19 (10.21)
Type of trauma	
Childhood or adulthood	35 (42.6%)
Both childhood and adulthood	44 (53.6%)
Ethnicity	
UK	80 (97.6%)
Other	2 (2.4%)
Education	
Basic (school/college)	68 (82.9%)
Higher education	14 (17.1%)
Employment	
Employed	23 (28.1%)
Unemployed	47 (57.3%)
Other	12 (14.6%)
Relationship status	
Married/cohabiting	23 (28.1%)
Other	59 (71.9%)
Living arrangements	
Living alone	35 (42.7%)
Living with other/s	46 (56.1%)
Psychotropic medication	
Yes	56 (68.3%)
No	13 (15.9%)

SD, standard deviation.

**Table 2:** Mean scores and standard deviations (SDs) of clinical scales

Measure	Minimum	Maximum	Mean (SD)
PCL-C			
Intrusion	5.0	25.0	18.1 (5.6)
Avoidance	9.0	35.0	23.5 (6.4)
Hyper-arousal	7.0	25.0	18.2 (4.4)
SCL-90			
Somatisation	0.2	3.9	2.2 (1.0)
Obsessive Compulsive	0.3	4.0	2.5 (0.9)
Interpersonal Sensitivity	0.1	4.0	2.5 (0.9)
Depression	0.5	4.0	2.7 (0.9)
Anxiety	0.2	4.0	2.5 (1.1)
Hostility	0.0	3.5	1.3 (0.9)
Phobic anxiety	0.0	4.0	2.4 (1.2)
Paranoid Ideation	0.0	3.7	2.0 (1.0)
Psychoticism	0.1	3.9	1.8 (0.9)
GSI	0.4	3.8	2.3 (0.8)
DES-90	2.9	85.7	34.0 (18.7)
RSES	0.0	27.0	9.3 (4.9)

SD, standard deviation; PCL-C, PTSD Checklist-Civilian Version; SCL-90, Symptom Checklist-90; GSI, Global Severity Index; DES, Dissociative Experiences Scales; RSES, Rosenberg self-esteem scale.

### EMS severity in interpersonal trauma survivors

Comparisons by means of independent sample *t*-test analyses between the clinical sample and control group on EMS are presented in Table 3. Statistically significant (*p*≤0.001) elevated EMS scores were reported in the interpersonal trauma group compared to the non-clinical group across all YSQ subscales. Our hypothesis that survivors of interpersonal trauma will present with elevated EMS compared to a non-clinical control group was supported. It was also quite interesting that the clinical group presented with elevated EMS scores across all 15 schemas and not solely the abuse-related schemas of Mistrust/Abuse, Defectiveness/Shame, or Vulnerability to Harm.

**Table 3:** Means (SDs) of YSQ subscales for clinical and control groups

YSQ subscale	Clinical mean (SD) (*n*=82)	Min/max scores	Control mean (SD) (*n*=78)	Min/max scores	Significance (df=156)
Domain: Disconnection					
Emotional Deprivation	22.6 (6.8)	5.0/30.0	4.0 (7.1)	0.0/28.0	*t*=16.7 *p*=0.001
Abandonment	19.2 (8.2)	5.0/30.0	7.0 (9.7)	0.0/30.0	*t*=8.5 *p*=0.001
Mistrust	22.8 (7.0)	6.0/30.0	6.0 (8.9)	0.0/29.0	*t*=13.3 *p*=0.001
Social Isolation	21.4 (7.5)	6.0/30.0	4.1 (8.1)	0.0/30.0	*t*=13.9 *p*=0.001
Defectiveness/Shame	20.7 (7.5)	5.0/30.0	3.1 (6.9)	0.0/30.0	*t*=15.3 *p*=0.001
Domain: Impaired Autonomy					
Failure	19.5 (8.6)	5.0/30.0	4.7 (9.4)	0.0/30.0	*t*=10.4 *p*=0.001
Dependence/Incompetence	16.4 (6.7)	5.0/37.0	3.8 (6.1)	0.0/27.0	*t*=12.4 *p*=0.001
Vulnerable to Harm	19.1 (7.1)	5.0/30.0	2.8 (6.1)	0.0/30.0	*t*=15.6 *p*=0.001
Enmeshment	9.8 (6.9)	5.0/30.0	1.8 (4.9)	0.0/28.0	*t*=8.3 *p*=0.001
Domain: Other-directedness					
Subjugation	18.4 (7.4)	5.0/30.0	3.4 (6.2)	0.0/25.0	*t*=13.8 *p*=0.001
Self-Sacrificing	22.1 (6.6)	5.0/30.0	9.3 (7.9)	0.0/30.0	*t*=11.1 *p*=0.001
Domain: Over-vigilance and inhibition					
Emotional Inhibition	17.0 (6.8)	5.0/30.0	3.8 (6.2)	0.0/25.0	*t*=12.7 *p*=0.001
Unrelenting standards	18.4 (6.7)	5.0/30.0	8.3 (9.1)	0.0/30.0	*t*=7.9 *p*=0.001
Domain: Impaired Limits					
Entitlement	9.7 (4.7)	5.0/25.0	4.9 (6.6)	0.0/28.0	*t*=5.2 *p*=0.001
Insufficient Self-Control	17.1 (6.9)	5.0/30.0	7.2 (8.6)	0.0/29.0	*t*=8.1 *p*=0.001
YSQ Total	273.6 (58.7)	160.0/390.0	74.0 (73.5)	0.0/298.0	*t*=18.6 *p*=0.001

SD, standard deviation; YSQ, Young Schema Questionnaire.

### Associations between EMS and psychopathology

The association between EMS and various forms of psychopathology was investigated by means of linear regression analysis with PCL-C, SCL-90, DES, and RSES as the predicting variables and the YSQ variables as the predictor variables. PCL-Intrusion was significantly predicted by Vulnerable to Harm (*B*=0.49, *t*=0.5, *p*=0.016). PCL-Avoidance was not predicted significantly (*p*≤0.05) by any of the EMS measures. PCL-Hyperarousal was significantly predicted by Vulnerable to Harm (*B*=0.51, *t*=2.9, *p*=0.005). PCL-total was significantly predicted by Vulnerable to Harm (*B*=0.51, *t*=3.0, *p*=0.004). The overall regression models applying all YSQ measures predicting individual psychopathology were applied, and the effect size of each is interpreted using Cohen (1992) guide of *r*=0.10 being small, *r*=0.30 being medium, and *r*=0.50 being a large effect size. The regression model significantly predicted PCL Avoidance (*F*=3.4, *p*=0.001), adjusted *R*^2^=0.323 and therefore explaining 32.3% of the variance with a medium effect size, Hyperarousal (*F*=2.7, *p*=0.001), adjusted *R*^2^=0.256, and therefore explaining 25.6% of the variance with a small–medium effect size, and Total PCL (*F*=3.4, *p*=0.001), adjusted *R*^2^=0.328, and therefore explaining 32.8% of the variance with a medium effect size. PCL Intrusion was not significantly predicted, with the regression model explaining just 6% of PCL Intrusion variance.

With regard to SCL subscales, SCL-Somatisation was significantly predicted by Vulnerable to Harm (*B*=0.61, *t*=0.40, *p*=0.001). SCL-Obsession/Compulsion was also significantly predicted by Vulnerable to Harm (*B*=0.71, *t*=5.1, *p*=0.001). SCL-Interpersonal Sensitivity was significantly predicted by Mistrust (*B*=0.38, *t*=3.1, *p*=0.003), and Defectiveness/Shame (*B*=0.43, *t*=2.9, *p*=0.005). SCL-Depression was significantly predicted by Emotional Deprivation (*B*=0.26, *t*=3.3, *p*=0.002), Defectiveness/Shame (*B*=0.55, *t*=4.3, *p*=0.001), Vulnerable to Harm (*B*=0.37, *t*=3.2, *p*=0.002), Dependence/Incompetence (*B*=0.30, *t*=2.7, *p*=0.009), and Abandonment (*B*= − 0.23, *t*= − 2.7, *p*=0.010). SCL-Anxiety was significantly predicted only by Vulnerable to Harm (*B*=0.55, *t*=3.8, *p*=0.001). SCL-Hostility was significantly predicted by Vulnerable to Harm (*B*=0.46, *t*=2.6, *p*=0.012) and Entitlement (*B*=0.37, *t*=2.6, *p*=0.012). SCL-Phobic Anxiety was significantly predicted by Vulnerable to Harm (*B*=0.66, *t*=4.3, *p*=0.001). SCL-Paranoid Ideation was significantly predicted by Mistrust (*B*=0.45, *t*=3.5, *p*=0.001) and Social Isolation (*B*=0.27, *t*=2.4, *p*=0.019). SCL-Psychoticism was significantly predicted by Vulnerable to Harm (*B*=0.47, *t*=3.3, *p*=0.002) and Defectiveness/Shame (*B*=0.47, *t*=3.0, *p*=0.004). Finally, SCL-GSI was significantly predicted by Vulnerable to Harm (*B*=0.57, *t*=4.7, *p*=0.001) and Defectiveness/Shame (*B*=0.34, *t*=2.5, *p*=0.014). All regression models were statistically significant in predicting pathology from the YSQ schemas. Using the adjusted *R*^2^, the models explained 26.3% of Somatisation variance (*p*=0.003), and 26.2% of Hostility variance (*p*=0.003) with a small-medium effect size. Regression models with large effect sizes explained 55.9% of Obsessive-Compulsive variance (*p*=0.001), 58.7% of Interpersonal Sensitivity variance (*p*=0.001), 69.5% of Depression variance (*p*=0.001), 50.8% of Anxiety variance (*p*=0.001), 45.9% of Phobic Anxiety variance (*p*=0.001), 55% of Paranoid Ideation variance (*p*=0.001), 53.1% of Psychoticism variance (*p*=0.001), and 66.3% of the GSI variation (*p*=0.001).

Dissociation as measured by DES was significantly predicted by Failure (*B*=−0.44, *t*=2.9, *p*=0.006), and Dependence/Incompetence (*B*=0.51, *t*=3.0, *p*=0.004). The overall model was statistically significant (*F*=2.7, *p*=0.003) and, with adjusted *R*^2^=0.257, explained 25.7% of the DES variance, a small-medium effect size. RSES total was not significantly predicted by any individual schemas, however the overall model was statistically significant (*F*=4.0, *p*=0.001) and, with adjusted *R*^2^=0.372, and explained 37.2% of the RSES variance, a medium-large effect size.

In line with our hypothesis, overall results indicate that different schemas are associated with different psychopathological features in people with interpersonal trauma. As shown in Table 4, schemas in the Impaired Autonomy domain were significantly associated with traumatic and dissociative symptomatology as well as obsessive compulsive symptomatology. Schemas in the Disconnection domain were predominantly associated with interpersonal sensitivity, depression, and paranoid ideation. Schemas in Disconnection and Impaired Autonomy domains were significantly associated with depressive symptomatology, interpersonal sensitivity, psychoticism, and general psychological distress. Although the pattern of associations between different psychopathological features and schemas appears to be complex, schemas in the domains of Disconnection and Impaired Autonomy formed significant associations with most psychopathological features in this study. Particularly the schema Vulnerable to Harm was found to be associated with the majority of psychopathology measures. Schemas in the domains of Other-directedness, Over-vigilance and Inhibition, and Impaired Limits did not form any significant associations with psychopathology variables in this study with the exception of Entitlement being associated with SCL-Hostility. Our hypothesis that different schemas will be associated with different psychopathological entities was confirmed.

**Table 4:** Patterns of significant associations between YSQ and PCL, SCL-90, DES and RSES

	PCL intrusion	PCL avoidance	PCL hyperarousal	PCL total	Somatisation	Obsessive compulsive	Interpersonal sensitivity	Depression	Anxiety	Hostility	Phobic anxiety	Paranoid ideation	Psychoticism	GSI	DES	RSES
Domain: Disconnection																
Emotional Deprivation								**+**								
Abandonment								**−**								
Mistrust							**+**					**+**				
Social Isolation												**+**				
Defectiveness/shame							**+**	**+**					**+**	**+**		
Domain: Impaired Autonomy																
Failure															**−**	
Dependence/incompetence								**+**							**+**	
Vulnerable to harm	**+**		**+**	**+**	**+**	**+**		**+**	**+**	**+**	**+**		**+**	**+**		
Enmeshment																
Domain: Other-directedness																
Subjugation																
Self-sacrificing																
Domain: Over-vigilance and inhibition																
Emotional inhibition																
Unrelenting standards																
Domain: Impaired Limits																
Entitlement										**+**						
Insufficient Self-Control																

PCL, PTSD Checklist; GSI, Global Severity Index; DES, Dissociative Experiences Scales; RSES, Rosenberg self-esteem scale.

+, indicates a significantly positive association; −, indicates a significantly negative association.

### Model-based cluster analysis

A cluster analysis was performed to identify two groups within the clinical group: low EMS and high EMS. Clusters are only meaningful and relevant with sufficient conceptual support; therefore, the technique is purely descriptive and atheoretical in nature. Based on previous research using clustering in EMS (e.g., Harding et al., 2012), the technique is justified in identifying differential elevations of EMS in the present sample. A maximum of 10 iterations were allowed to identify significantly different clusters, and the process was completed after four iterations. Subsequently, an ANOVA compared the group differences between these clusters. The clusters were statistically significantly different (*p*<0.025) for all the YSQ sub-scales except for Emotional Deprivation, Self-Sacrifice, and Entitlement. All other scales were significant at *p*<0.007. These non-significant scales, when compared to the control group mean, have high means in both the low and high EMS groups. This suggests that for these subscales a ceiling effect may be evident, in which most participants with interpersonal trauma have scored so highly that a difference is not detected between clusters. This in itself indicates some importance of these schemas to interpersonal trauma.

**Fig. 1:**
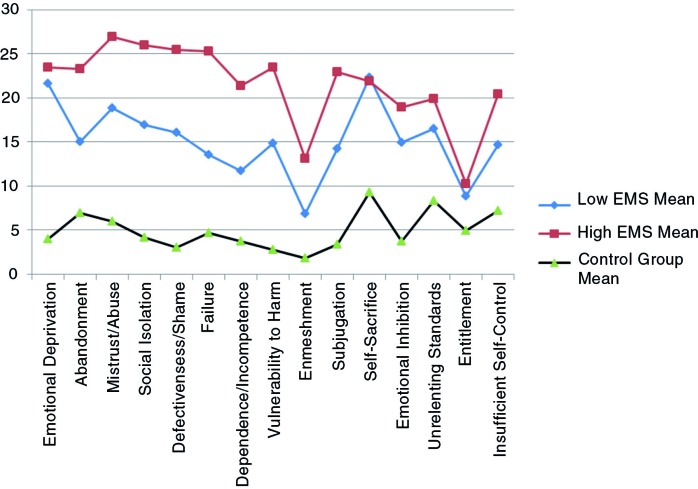
Mean YSQ scores for low and high early maladaptive schemas (EMS) clusters and control group.

Cluster analysis showed that the two clinical sub-groups were differentiated by their severity on the YSQ sub-scales. The first cluster, low to moderate EMS (*n*=41), exhibited lower scores on each of the schema sub-scales. The second cluster, high EMS (*n*=35), exhibited higher scores on each of the schema sub-scales. Both clusters scored higher than the control group across the sub-scales. Overall, our hypothesis that groups of people with interpersonal trauma differed in relation to EMS severity patterns was supported. However, the patterns of certain EMS elevation in either clusters were not observed (Fig. 1).

### Cluster validation in terms of EMS severity

To validate the two clusters in terms of EMS severity, we statistically compared the two clusters on all YSQ subscales. Results from this analysis are presented in Table 5. With the exception of the Self-Sacrifice schema, those on the high EMS cluster presented with significantly (*p*≤0.025) elevated EMS scores. Although a higher mean of Self-Sacrifice presented in the low EMS cluster compared with the high EMS cluster, these differences were not statistically significant (*p*≥0.025). Non-statistically significant differences between the two clusters were also observed in the Emotional Deprivation and Entitlement schemas. Overall results indicate that interpersonal trauma is associated with various levels of schema severity across most different schema domains.

**Table 5:** Interpersonal trauma subgroups means on EMS subscales

YSQ items	Low EMS *n*=41 Mean (SD)	High EMS *n*=35 Mean (SD)	*t* (df=74)	*p*
Domain: Disconnection				
Emotional Deprivation	21.3 (6.6)	24.0 (7.0)	1.7	0.092
Abandonment	15.5 (7.5)	23.2 (7.2)	4.5	0.001
Mistrust/Abuse	19.2 (7.2)	26.9 (3.7)	5.7	0.001
Social Isolation	17.0 (7.1)	26.4 (4.4)	6.8	0.001
Defectiveness/Shame	16.0 (6.5)	25.9 (4.6)	7.5	0.001
Domain: Impaired Autonomy				
Failure	13.6 (6.4)	25.9 (5.6)	8.8	0.001
Dependence/Incompetence	12.4 (5.7)	21.1 (4.8)	7.2	0.001
Vulnerability to Harm	15.0 (6.6)	23.9 (3.8)	7.1	0.001
Enmeshment	7.1 (3.0)	13.2 (8.7)	4.2	0.001
Domain: Other-directedness				
Subjugation	14.5 (6.1)	23.1 (6.4)	6.0	0.001
Self-Sacrifice	22.5 (6.0)	21.7 (7.3)	**−**0.5	0.604
Domain: Over-vigilance and inhibition				
Emotional Inhibition	14.7 (6.2)	19.5 (6.8)	3.2	0.002
Unrelenting Standards	16.2 (6.2)	20.3 (6.6)	2.8	0.007
Domain: Impaired Limits				
Entitlement	9.2 (4.3)	10.0 (4.9)	0.7	0.467
Insufficient Self-Control	15.0 (5.8)	20.3 (6.7)	3.7	0.001
Total YSQ	229.3 (33.8)	325.4 (33.6)	12.4	0.001

YSQ, Young Schema Questionnaire; EMS, early maladaptive schemas.

### Cluster differences in clinical scales

The two clusters were compared on levels of traumatic, general, and dissociative symptomatology as well as self-esteem levels. Table 6 summarises the results of this comparison. The high EMS group exhibited statistically significant (*p*≤0.025) more severe traumatic avoidance and hyperarousal symptomatology as measured by PCL-C subscales and total. The high EMS group also exhibited significantly (*p*≤0.025) more severe Obsessive–Compulsive, Interpersonal Sensitivity, Depression, Anxiety, Hostility, Paranoid Ideation, Psychoticism, and general distress (GSI) compared with the low EMS group as measured by SCL-90. The low EMS group exhibited a higher mean in SCL-somatisation subscale, but this difference was not statistically significant. Finally, the high EMS group exhibited significantly (*p*≤0.025) lower self-esteem as measured by RSES. The high EMS group exhibited more severe dissociation as measured by DES; however, this result was only moderately significant at *p*=0.031. Overall, our hypothesis that high EMS will be associated with more severe traumatic and other types of symptomatology, more severe dissociation, and lower self-esteem was supported.

**Table 6:** EMS subgroups on measures of traumatic and general psychopathology, dissociation and self-esteem

	YSQ			
			
Clinical measures	Low EMS *n*=41 Mean (SD)	High EMS *n*=35 Mean (SD)	*t* (df=74)	*p*
PCL-C				
PCL Intrusion	16.5 (6.0)	19.4 (4.7)	2.3	0.024
PCL Avoidance	21.0 (6.5)	26.0 (5.4)	3.6	0.001
PCL Hyperarousal	17.0 (4.5)	19.5 (4.2)	2.5	0.014
PCL Total	54.4 (14.2)	64.9 (11.3)	3.5	0.001
SCL-90				
Somatisation	1.8 (1.0)	2.4 (0.9)	**−**0.9	0.371
Obsessive Compulsive	2.0 (0.9)	2.9 (0.6)	5.3	0.001
Interpersonal Sensitivity	1.9 (1.0)	3.0 (0.5)	6.0	0.001
Depression	2.2 (0.9)	3.2 (0.5)	6.1	0.001
Anxiety	2.0 (1.2)	3.0 (0.7)	4.6	0.001
Hostility	1.0 (0.8)	1.6 (0.9)	2.9	0.005
Phobic Anxiety	1.9 (1.3)	2.9 (0.7)	4.1	0.001
Paranoid Ideation	1.4 (0.9)	2.6 (0.6)	6.8	0.001
Psychoticism	1.3 (0.8)	2.3 (0.7)	5.8	0.001
GSI	1.8 (0.8)	2.7 (0.5)	5.8	0.001
DES	28.8 (19.9)	38.1 (15.8)	2.2	0.031
RSES	11.3 (4.5)	7.1 (4.0)	**−**4.2	0.001

YSQ, Young Schema Questionnaire; SD, standard deviation; EMS, early maladaptive schemas; PCL-C, PTSD Checklist-Civilian Version; SCL-90, Symptom Checklist-90; GSI, Global Severity Index; DES, Dissociative Experiences Scales; RSES, Rosenberg self-esteem scale.

## Discussion

The broad aim of our study was to investigate the association between EMS and common forms of psychopathology in a sample of women with a history of interpersonal trauma. We have hypothesised that survivors of interpersonal trauma will present with elevated EMS scores compared to a non-clinical control group. We have also hypothesised that different schemas will be associated with different psychopathological entities and that different subgroups of interpersonal survivors would be present in our sample, with subgroups displaying different profiles of schema severity elevations. It was found that survivors of interpersonal trauma displayed elevated EMS scores across all 15 schemas and not solely in the abuse-related schemas of Mistrust/Abuse, Defectiveness/Shame, or Vulnerability to Harm. These particular schemas are associated with abuse according to Young et al. (2003). It was also found that high levels of EMS were associated with more severe traumatic stress and other types of psychopathology, more severe dissociation, and lower self-esteem. Although the pattern of associations between different psychopathological features and schemas appears to be rather complex, schemas in the domains of Disconnection and Impaired Autonomy formed significant associations with most psychopathological features in this study. Schemas in the domains of Other-directedness, Over-vigilance and Inhibition, and Impaired Limits did not form any significant associations with psychopathology variables in this study with the exception of Entitlement and SCL-Hostility.

Consistently with Harding et al. (2012), our findings indicate that women with interpersonal trauma comprise a heterogeneous group. This consists of subgroups that can be distinguished meaningfully by EMS severity, rather than the overall shape or distribution of the EMS profile, with the relative schema elevations being distributed across two of the five schema domains. These results suggest that interpersonal trauma survivors are distinguished primarily by a generalised elevation of their maladaptive schemas, rather than a unique schema profile comprised of specific schemas. The results also indicate that schemas may function more as a global measure of general cognitive vulnerability (e.g., McGinn et al., 2005). Within a general cognitive vulnerability model, the severity level of EMS may have less specificity with regard to various psychopathological outcomes. However, a strong profile was formed in the domains of Disconnection and Impaired Autonomy, where both presented with strong associations with psychopathological entities investigated in this study.

Considering that schemas were found to form significant associations with various forms of psychopathology in this study, this finding is in support of cognitive theories of psychopathology such as the cognitive model of PTSD (e.g., Ehlers & Clark, 2000) or depression (Beck, Rush, Shaw, & Emery, 1979) in people with interpersonal trauma. In particular, and in line with previous research in the area (e.g., anxiety; Lumley & Harkness, 2007) the schema vulnerable to Harm, formed significant associations with a number of different psychopathological features. This schema refers to beliefs that others may be expected to intentionally hurt, abuse, or humiliate, and an exaggerated belief that catastrophe or harm can unpredictably strike at any time (Young & Brown, 1994b). Our results support Young's et al. (2003) hypothesis that the schema Vulnerable to Harm is highly associated with early traumatic or victimisation experiences of an interpersonal nature.

Our study had a number of limitations. Our sample was homogeneous and consisted solely of people with interpersonal trauma. Although our design was strengthened by the inclusion of a control group, it would have been interesting to compare schema profiles and severity of psychopathology between people with interpersonal and non-interpersonal trauma in the same population. There is evidence to suggest that different schema profiles are active in different trauma groups. Although we found that Vulnerability to Harm and Enmeshment were predictive of traumatic pathology in people with interpersonal trauma, Price (2007), in a sample of men and women with PTSD following work-related trauma, either interpersonal or non-interpersonal, found that four schemas (Defectiveness, Dependency, Enmeshment, and Failure) significantly predicted PTSD status. Further to this, psychopathology resulting from certain types of interpersonal trauma may be more strongly associated with certain schemas. For example, Harding et al. (2012) in a sample of CSA survivors found that Schemas of Mistrust/Abuse, Vulnerability to Harm, and Emotional Deprivation contributed most to distinguishing women with a diagnosis of PTSD whereas in our study only the schemas of Vulnerability to Harm and Enmeshment were predictive of PTSD severity. Furthermore, we have had limited information in relation to trauma characteristics such as severity or duration of trauma, and we have used self-rated scales. Although one can argue that these factors may be mediating or confounding the relationship between EMS and psychopathology, there is some evidence to suggest (e.g., Bak-Klimek et al., 2013) that trauma factors may not be associated with the severity of pathology in survivors of interpersonal trauma. In addition, as the control group data were extracted from a previous study, trauma history data were not available in this group. A more meaningful comparison would be against a control group without a trauma history from the same clinical population. Given the cross-sectional nature of our data and the possibility of affective bias in responding, it is not possible to draw any conclusions with regard to the association between severity of EMS and pathology development or maintenance of symptomatology. It is important that future research employs prospective designs to determine the directionality of associations between EMS and psychopathology.

There were also significant negative associations identified between SCL-Depression and YSQ-Abandonment, and between DSES and YSQ-Failure. There are a number of reasons as to why this may be the case. Lower scores on Abandonment may predict greater scores on Depression in this sample because of the heterogeneity of trauma exposures coupled with the small sample size. Some traumas may include an intentional form of abandonment, for example, domestic abuse and childhood neglect, whereas other traumas might involve non-intentional abandonment such as bereavement. The differential effect of these on the development of a depressive schema might not be able to be captured by this study design and warrants further investigation. It is also unclear why it was found that lower scores on YSQ-Failure predicted greater dissociation symptoms. There may be a mediating variable that has not been captured by this analysis that could explain these incongruous results. Dissociation is more strongly associated with severe and chronic abuse rather than single event traumas, so again it is possible that the schema profile for Failure develops differently from traumas experienced in childhood and those in adulthood. Further research unpicking these relationships comparing child and adult trauma would shed more light on these findings.

Notwithstanding its limitations, this is the first study to investigate the association between EMS and various forms of common psychopathology in a group of survivors of interpersonal trauma. The study contributes to our understanding of maladaptive and enduring cognitive schemas about the self, world, and others among survivors of interpersonal trauma. Our findings support the usefulness of cognitive behavioural interventions that target schemas in the domains of Disconnection and Impaired Autonomy in an effort to modify existing core beliefs and decrease associated symptomatology in adult survivors of interpersonal trauma.
